# Prevalence and Sociodemographic Predictors of Mental Health in a Representative Sample of Young Adults from Germany, Israel, Poland, and Slovenia: A Longitudinal Study during the COVID-19 Pandemic

**DOI:** 10.3390/ijerph19031334

**Published:** 2022-01-25

**Authors:** Joy Benatov, Dominika Ochnik, Aleksandra M. Rogowska, Ana Arzenšek, Urša Mars Bitenc

**Affiliations:** 1 Department of Special Education, University of Haifa, Haifa 3498838, Israel; jbentov2@gmail.com; 2 Faculty of Medicine, University of Technology, 40-555 Katowice, Poland; 3 Institute of Psychology, University of Opole, 45-052 Opole, Poland; arogowska@uni.opole.pl; 4 Faculty of Management, University of Primorska, 6101 Koper, Slovenia; ana.arzensek@fm-kp.si; 5 Department of Psychology, Faculty of Mathematics, Natural Sciences and Information Technologies, University of Primorska, 6101 Koper, Slovenia; ursa.mars@upr.si

**Keywords:** mental health, young adults, COVID-19, coronavirus-related post-traumatic stress disorder, anxiety, depression, perceived stress, longitudinal study, cross-national study

## Abstract

The aim of this cross-national longitudinal study was to evaluate the prevalence and sociodemographic predictors of mental health indicators (coronavirus-related post-traumatic stress disorder (PTSD), perceived stress, anxiety, depression, and suicidal/self-harm ideation) during the coronavirus disease-2019 (COVID-19) pandemic in a three-month period among representative samples of young adults from Germany, Israel, Poland, and Slovenia. The participants were 1724 young adults between 20 and 40 years of age (M = 30.74, SD = 5.74). The first measurement (T1) was in February 2021 and the second (T2) was in May–June 2021. The samples were representative of young adults in each country: Germany (*n* = 418, 24%), Israel (*n* = 428, 25%), Poland (*n* = 446, 26%), and Slovenia (*n* = 431, 25%). Women constituted 54% (*n* = 935) of the total sample. The mental health indicators were coronavirus-related PTSD measured by PCL-S, perceived stress (PSS-10), anxiety (GAD-7), depression (PHQ-8), and suicidal ideation (PHQ-9). The participants completed an online questionnaire that also included a physical activity (PA) measurement and sociodemographic variables. The Pearson’s χ^2^ independence test was used for prevalence comparisons and McNemar’s χ^2^ was used for longitudinal changes, whereas generalized estimating equations (GEEs) were used for the predictors of change in mental health indices. Significant differences were found between countries in each mental health dimension in both T1 and T2, with moderate effect sizes for coronavirus-related PTSD and suicidal ideation. The highest rate of PTSD and depression risk was in Germany, the highest rates of stress and anxiety risk were in Poland, and there was insufficient PA in Slovenia. The anxiety, depression, and suicidal ideation rates were the lowest in Israel and Slovenia. Israeli participants reported the lowest rate of coronavirus-related PTSD among the other countries in T1 and T2. Significant decreases in coronavirus-related PTSD and stress were observed during T2 compared to T1 in the total sample. There was no change in the risk of anxiety, depression, or suicidal ideation. Being single was a predictor of changes in all mental health indices. Having children was a risk factor for coronavirus-related PTSD and high stress. Being a student was a predictor of depression and suicidal ideation. A younger age (20–29 years) predicted coronavirus-related PTSD risk, whereas female gender predicted high stress. The mental health indices improved over time or remained stable. The groups that are most prone to mental health problems were single individuals, students, and parents in young adulthood across all countries. Future intervention programs for young adults should consider these factors when prioritizing, planning, and implementing such programs.

## 1. Introduction

The novel coronavirus disease-2019 (COVID-19) appeared in December 2019 and rapidly spread across the globe, bringing about an immense health, economic, and social crisis [1]. The COVID-19 pandemic was declared on 11 March 2020 and brought suffering to those infected by the virus and their loved ones. Furthermore, the general population has been exposed to stressors due to the fear of being infected and the preventive restrictions imposed, which included social distancing, isolation, quarantine, and loss of employment [2]. Such stressors may cause substantial mental health difficulties, especially among vulnerable populations, including the elderly, individuals with chronic diseases, and low-income families [3,4].

Young adulthood is a developmental period with unique biological, psychological, and social risk factors that may be affected by the pandemic, even though this age group is less susceptible to COVID-19 infection [5,6,7]. This developmental period is characterized by distinctive challenges that include attaining post-secondary education, developing a career path, forming romantic relationships, gradually reaching financial and emotional independence from parents, and often transitioning to parenthood [8,9]. A large-scale longitudinal study conducted in the U.K. reported a rise in mental health distress between 2018–2019 and April 2020, particularly among 18–24-year-olds [5]. An additional report from the U.K. Household Longitudinal Study reported that psychological distress increased one month into lockdown, particularly among women and young adults [6]. These studies point to young adults as a potentially vulnerable group to mental health deterioration during the pandemic. Studies from different countries reporting on college students’ concerns during the pandemic have found them to include educational concerns (e.g., adjustment to online teaching and motivational issues), social concerns (e.g., lack of social interaction with peers and family), employment stability/financial concerns (e.g., maintaining a job and a lack of professional opportunities), mental health concerns (e.g., experiencing symptoms of anxiety and depression), and health concerns [10,11,12]. These different sources of stress can affect overall mental health. 

Upon the eruption of the pandemic, the professional mental health community warned about the potentially devastating effects on mental health [13,14]. Since then, many studies reporting rates of mental health difficulties during the pandemic have been published, showing a complex pattern of results. A meta-analysis of 65 longitudinal cohort studies comparing mental health before versus during the COVID-19 pandemic reported a significant increase in both anxiety and depression during the outbreak of the pandemic (March–April 2020), followed by a decline to near pre-pandemic levels on most measures except depression by mid-2020 [15]. Similarly, a review conducted by Aknin and colleagues [16] on mental health during the first year of the COVID-19 pandemic found evidence that anxiety, depression, and distress increased in the early months of the pandemic, after which some studies reported a decline to pre-pandemic levels, while other studies reported persistently elevated levels of mental distress compared to pre-pandemic levels [16]. A longitudinal study conducted in Australia during the pandemic showed that the number of participants with clinical levels of depressive, anxiety, or insomnia symptoms did not differ statistically between April and September 2020, whereas well-being and stress showed a slight improvement [17].

Similarly, a longitudinal study conducted in the U.K. reported a sharp increase in mental health difficulties in the initial stages of the COVID-19 outbreak (March–April 2020), which diminished several months afterward (June 2020) [18]. However, despite the decline, mental health problems remained elevated compared to pre-pandemic levels [18]. Taken together, these studies point to an increase in mental health difficulties in the early stages of the pandemic and adaptation responses to some degree in the following months.

Several patterns of responses to potentially traumatic life events have been put forward in the literature, including chronic dysfunction, minimal-impact resilience (consistent low levels of mental health symptoms or distress), and emerging resilience or recovery (a gradual recovery to mental health adjustment following a period of struggle) [19]. The pattern of mental health difficulties following the pandemic found in Robinson’s meta-analysis reflects an emerging resilience response, with an acute response to the distressing event at the beginning of the pandemic followed by a period of gradual psychological adaptation [15]. However, it remains challenging to predict the evolution of mental health difficulties over time as the pandemic unfolds, as they depend on various factors such as past and present stress, degree of exposure, available social and economic resources, demographic variables, and personality traits [19]. The prevalence of mental health difficulties varies by country and the state of the pandemic at a specific time point. For example, Ochnik et al. [7] compared the mental health of university students in nine countries. They revealed that Turkish students reported the highest depression levels (perhaps due to economic burden and a high unemployment rate), while Rogowska et al. [20] showed an increase in perceived stress between the first and second wave of the pandemic in Poland.

There is still limited information about mental health changes during the COVID-19 pandemic. Very few studies have provided within-subject longitudinal reports from cross-national sampling. Thus, the main goal of the current study was to evaluate the rates and changes in the rates of coronavirus-related post-traumatic stress disorder (PTSD), perceived stress, anxiety, depression, and suicidal/self-harm ideation during the pandemic among representative samples of young adults from four countries (Germany, Israel, Poland, and Slovenia). In addition, we wanted to examine whether sociodemographic variables such as age, gender, place of residence, student status, relationship status, and having children predict changes in mental health indices. Factors such as younger age, female gender, being single, and having children were associated with worse mental health during the COVID-19 pandemic [21,22,23].

## 2. Materials and Methods

### 2.1. Study Design

The ARIADNA panel provided the data collection in Germany, Israel, Poland, and Slovenia. To address potential sources of bias, the samples in each country were representative of gender, student status, and employment status. The inclusive criterion was an age between 20 and 40 years old. The participants were not directly financially rewarded; however, they were enrolled in the reward system. The participants could gain points by fulfilling the questionnaires, which could be exchanged for prizes, cash, or charity donations. 

In Germany, participants were recruited from 11 federated states; in Poland, from all 16 voivodeships; in Slovenia, from East and West Slovenia; and in Israel, from six main districts and the Judea and Samaria area. A detailed description can be found in Supplementary Table S1.

**Figure 1:**
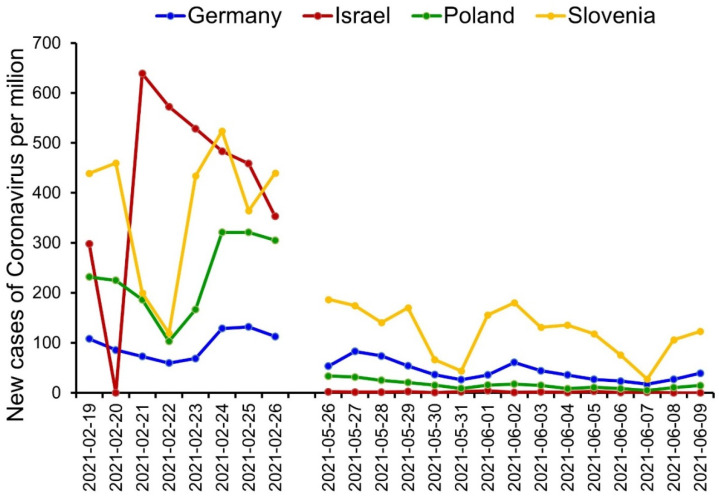
Number of new cases of COVID-19 infections per million citizens in four countries (Germany, Israel, Poland, and Slovenia) during the data collection in T1 (19–29 February 2021) and T2 (26 May–9 June 2021). Source: Johns Hopkins University Center for Systems Science and Engineering (CSSE) COVID-19 Data [24].

This longitudinal study was conducted in two waves over a three-month period. The first measurement of the study (T1) was conducted between 19 and 26 February 2021, whereas the second measurement (T2) was conducted between 26 May and 9 June 2021. A significantly higher number of new cases of COVID-19 were present during T1 as compared to T2. The most cases of COVID-19 in T1 and the fewest cases in T2 were found in Israel when comparing the four countries. Some differences between countries in the number of new cases per million were noted during both timepoints of the study [24] (see Figure 1 for more detail). 

The stringency index (SI) [25] is a measure of the level of restriction during the COVID-19 pandemic (ranging from 0 to 100) that consists of nine indicators: the cancellation of public events, restrictions on public gatherings, closures of public transport, stay-at-home requirements, public information campaigns, internal traffic restrictions, and international travel control.

During T1, the mean SI was 75.35 in Germany (ranging from 75.00 to 77.78, SD = 0.98), 66.90 in Israel (ranging from 62.96 to 78.70, SD = 7.29), 71.30 in Poland (unchanged), and 61.11 in Slovenia (unchanged). During T2, the mean SI was 70.80 in Germany (ranging from 67.59 and 75.00, SD = 3.17), 38.89 in Israel (ranging from 29.63 to 52.78, SD = 11.74), 56.48 in Poland (ranging from 53.70 and 62.04, SD = 2.57), and 35.19 in Slovenia (unchanged). The mean of total vaccinations per hundred was 6.49 (SD = 0.47), 87.62 (SD = 3.67), 13.54 (SD = 15.93), and 7.53 (SD = 0.53) in Germany, Israel, Poland, and Slovenia, respectively, during T1. In contrast, during T2, the mean of total vaccinations per hundred was 62.83 (SD = 3.82), 122.41 (SD = 0.15), 55.82 (SD = 3.42), and 54.73 (SD = 0.3.86) in Germany, Israel, Poland, and Slovenia, respectively.

Initially, there were 2951 participants in T1. During T2, 1724 participants responded. The response rate was 58.42% in T2. One observation from T2 was excluded due to an anomaly in the pattern of detection. The final sample consisted of 1723 participants across Germany, Israel, Poland, and Slovenia.

In the online survey questionnaire, answering all of the questions was required to continue with the survey; therefore, the participants could not omit any questions. Additionally, respondents who decided not to reveal their gender were excluded from statistical analyses concerning gender (*n* = 6). There was no time limit for fulfilling the questionnaire. The participants could stop at any moment and return at any time to finish the survey. The average time of data collection was 21.52 min (SD = 136.75). The participants in each country filled in the survey in their native language. According to the cross-cultural adaptation standards [26,27], experts from various countries translated the survey questions from English to the languages of the participating countries.

### 2.2. Participants

The participants were 1723 young adults, ranging in age from 20 to 40 years (M = 30.74, SD = 5.74). The total sample represented adults from Germany (*n* = 418, 24%), Israel (*n* = 428, 25%), Poland (*n* = 446, 26%), and Slovenia (*n* = 431, 25%). A minimal sample size of 159 for each country was determined using G*Power software [28] with regard to 209 χ^2^ contingency tables, with *p* < 0.05 (two-tailed) and a 95% CI. Therefore, we aimed to gather samples of 200 participants for each age group (20–29 and 30–40 years of age) in each of the four countries. Table 1 demonstrates the percentage and number of people in each sociodemographic category. The sample was divided into younger adults (20–29 years; *n* = 840, 49%) and adults (30–40 years; *n* = 883, 51%). The majority of the participants were women (*n* = 935, 54%), lived in towns or cities (*n* = 1297, 75%), declared their relationship status as coupled (*n* = 1218, 71%), and were child-free (*n* = 1001, 58%). Most individuals in the study were currently employed (*n* = 1324, 77%) and were not students at the time of completing the survey (*n* = 1303, 76%). However, significant differences were present between the four countries in terms of place of residence, relationship status, having children, current student status, and employment status, as the Pearson’s χ^2^ contingency table indicates (Table 1).

### 2.3. Measurements

#### 2.3.1. Coronavirus-Related PTSD 

Coronavirus-related PTSD was assessed using the 17-item PTSD Checklist-Specific Version (PCL-S) [29] on a five-point Likert scale ranging from 1 to 5 (1 = not at all; 2 = a little bit; 3 = moderately; 4 = quite a bit; 5 = extremely) with a total score ranging from 17 to 85. For the purposes of conducting the χ^2^ independence test and logistic regression, coronavirus-related PTSD was dichotomized as follows: 0 = no risk (no severity or some symptoms, 17–29), and 1 = risk (moderate or high severity, 30–85).

Even though PCL-S is based on the Diagnostic and Statistical Manual of Mental Disorders, fourth edition (DSM-4) [30], we decided to use this particular version to be sure that we evaluated the risk of PTSD related to a specific stressful event, which, in this case, was the lockdown due to the COVID-19 pandemic. Additionally, the newest version, PLC-5 [31], which is based on the DSM-5 [32], is considered to be very similar to PCL-S, but does not refer to a specific stressful experience. Moreover, it has been suggested by the National Centre for PTSD to follow DSM-4 recommendations to evaluate the change [31]. In this study, the participants evaluated how much they were bothered by the specific problem of COVID-19 in the past month. In each of the 17 items, we added the following expression: “A stressful experience from the COVID-19 lockdown” (e.g., Item 1: Repeated, disturbing memories, thoughts, or images of a stressful experience from the COVID-19 lockdown). Cronbach’s α for COVID-19-related PCL-S in this study was 0.96 at both T1 and T2 in the total sample.

#### 2.3.2. Perceived Stress

The Perceived Stress Scale (PSS-10) was used to measure whether the respondents appraised the situation in their life as stressful [33]. Perceived stress is related to the subjective assessment of events occurring in one’s life [34] and evaluates how unpredictable, uncontrollable, and overloaded individuals find their lives [33]. The 10 items of the PSS-10 refer to the frequency of stressful events that occurred in the month preceding the study. Items are evaluated on a five-point scale (0 = never to 4 = very often). Cronbach’s α for PSS-10 in this study was 0.83 in both T1 and T2.

#### 2.3.3. Anxiety

The Generalized Anxiety Disorder (GAD-7) scale [35] was utilized to measure anxiety risk. GAD-7 is a seven-item self-reported measure designed to screen for symptoms following the criteria of the Diagnostic and Statistical Manual of Mental Disorders, fifth edition (DSM-5) [32]. Generalized anxiety disorder (GAD) is depicted as a persistent and excessive worry about various issues and relates to anxiety as a state [35]. People rate how often they experienced anxiety symptoms for the two weeks preceding the study on a four-point scale (0 = not at all; 1 = several days; 2 = more than half the time; 3 = nearly every day). The GAD-7 scale ranges from 0 to 21. Anxiety disorder risk is indicated when the scores equal 10 points or above [35]. In order to conform with the χ^2^ independence test and logistic regression requirements, GAD-7 was dichotomized as follows: 0 = no risk, for GAD-7 below 10, and 1 = risk, for GAD-7 scores equal or above 10. Cronbach’s α for GAD-7 in this study was 0.94 in T1 and 0.95 in T2.

#### 2.3.4. Depression and Suicidal Ideation

The Patient Health Questionnaire (PHQ-8) [36] was utilized to evaluate depression risk. The PHQ-8 consists of eight items, conforming to the DSM-5 diagnostic criteria [32]. The symptoms include depressed mood, loss of interest in most or all activities, loss of energy, or feelings of worthlessness [36]. 

Participants use a Likert-type response scale ranging from 0 = not at all to 3 = nearly every day. The PHQ-8 total scores range from 0 to 24. A cut-off score of 10 or above indicates screening for major depressive disorder risk [36]. Due to the requirements of further statistical analysis with the use of the χ^2^ independence test and logistic regression, the PHQ-8 was dichotomized as follows: 0 = no risk (PHQ-8 < 10) and 1 = risk (PHQ-8 > 10). The internal consistency reliability in this study, measured by Cronbach’s α, was 0.93 in T1 and 0.94 in T2. 

The last item of the PQH-9 [37] was introduced to evaluate suicidal/self-harm ideation. The PQH-9 is an extended version of the PQH-8. Question 9 is used to screen the presence and duration of suicidal ideation. The participants answer on a Likert scale ranging from 0 to 4 (0 = not at all; 1 = several days; 2 = more than half the days; 3 = nearly every day). Suicidal ideation reported on the PHQ-9 is a strong predictor of suicide attempts and deaths [38].

#### 2.3.5. Physical Activity 

The level of physical activity (PA) was evaluated by two questions. The first question was: “How many days a week did you exercise physically or pursue sports activities at home or away from home, at the university, in clubs, or at the gym, in the last month?” [39]. The participants marked their answer on an eight-point scale (from 0 = not one day to 7 = seven days a week). The second question was open-ended: “How many minutes a day (on average) did you practice?”. The PA level was calculated by the multiplication of the numbers of days a week and minutes per day. Regarding the World Health Organization’s (WHO) recommendation [40], we divided the total sample into two groups: Sufficient (PA > 150 min weekly) and insufficient (PA < 150 min weekly).

#### 2.3.6. Sociodemographic Data 

Sociodemographic data included questions regarding gender, age (20–30 and 31–40 years), place of residence (village, town, or city), status (student or not a student), employment status (employed or unemployed), relationship status (coupled or single), and having children (having children or child-free). 

### 2.4. Statistical Analyses

The descriptive statistics included frequencies (number of participants and proportion in percentage) of sociodemographic variables, specifically, binary categories of gender, age, place of residence, student status, employment status, relationship status, and having children. The prevalence of PTSD, perceived stress, anxiety, PA, depression, and suicidal ideation was assessed during T1 (February 2021) and three months later during T2 (May–June 2021). A series of contingency tables were used to perform Pearson’s χ^2^ independence tests, comparing frequencies of participants in particular mental health dimensions in four countries: Germany, Israel, Poland, and Slovenia. Cramer’s V was used to calculate the effect size for the χ^2^ test.

McNemar’s χ^2^ test was used to examine the hypothesis that the proportions of mental health problems change (increase or decrease) over time in the adult sample during the COVID-19 pandemic. McNemar’s *χ*^2^ is a nonparametric analysis examining marginal homogeneity in a dichotomous variable measured at two different time points [41]. The marginal homogeneity is confirmed if the row and corresponding column marginal frequencies are equal in a 2 × 2 contingency table. As such, the McNemar’s χ^2^ test hypothesis checks whether the average difference between two time points is different from zero.

Generalized estimating equations (GEEs) were used in the study to account for correlated observations in longitudinal data, considering the dependence that occurs with multiple observations per person [41]. Although McNemar’s χ^2^ test can provide population-averaged estimates of change over time, GEEs can calculate both population-averaged as well as subject-specific estimates. The GEE approach enables correction for a dependency of observations within individuals over time by choosing a correlation structure. The independent working correlation structure implies that within-person correlations between all measurements are equal to zero. This seemed to be the best option given the fact that adjustments were made for within-subject correlations by the modeling of changes over time rather than absolute values at different time points [41]. GEEs with a robust standard error, binary logistic model, and independent working correlation structure were used in the study to assess the longitudinal change between T1 and T2 in such indices of mental health as coronavirus-related PTSD (no risk = 0, risk = 1), perceived stress (low = 0, high = 1), anxiety (no risk = 0, risk = 1), depression (no risk = 0, risk = 1), and suicidal ideation (no = 0, yes = 1). Model 1 was performed separately for PTSD, perceived stress, anxiety, depression, and suicidal ideation. When one variable was included in Model 1 as a dependent variable (e.g., anxiety), all other mental health indices were considered as confounding time-varying covariates (e.g., coronavirus-related PTSD, perceived stress, depression, and suicidal ideation). PA was included as an additional confounding time-varying covariate, due to significant associations with mental health indices.

In Model 2, sociodemographic variables were additionally included in previous GEE models to examine the adjusted effect of sociodemographic predictors on changes over time on mental health. Model 2 was performed separately for PTSD, perceived stress, anxiety, depression, and suicidal/self-harm ideation in T1 and T2, which were considered dependent variables. Each model included the following sociodemographic variables as factors (time-invariant covariates): gender (men = 0, women = 1), age (adults = 0 for 30–40 years of age, younger adults = 1 for 20–29 years of age), student status (no = 0, yes = 1), employment status (unemployed = 0, employed = 1), relationship status (coupled = 0, single = 1), and having children (no = 0, yes = 1). The place of residence was excluded from the analysis because the Israeli sample comprised only 38 participants living in rural areas; thus, the results could be biased (Table 1). Similar to Model 1, all other mental health variables were also included in each analysis as confounders. As the analysis requires a distinction between predictor and outcome variables, each model used the change in mental health as an outcome variable. All statistical analyses were conducted using SPSS v. 26.0.

## 3. Results

### 3.1. Prevalence of Mental Health in the Four Countries

**Hypothesis** **1** **(H1).** *The prevalence of mental health indicators will differ across Germany, Israel, Poland, and Slovenia among young adults during T1 and T2.*

**Figure 2:**
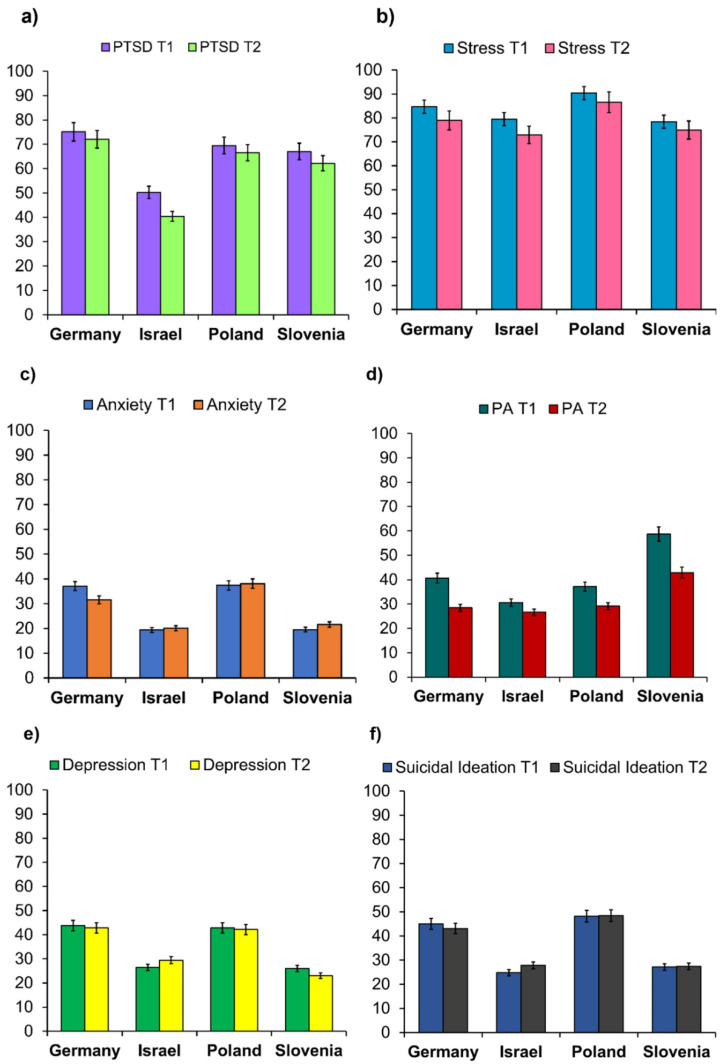
Percentage of young adults with: (**a**) coronavirus-related post-traumatic stress disorder (PTSD) risk; (**b**) perceived stress (PSS-10); (**c**) anxiety risk (GAD-7); (**d**) insufficient level of physical activity (PA < 150 min per week); (**e**) depression risk (PHQ-9); and (**f**) suicidal/self-harm ideation (Question 9 of the PHQ-9). Error bars are 95% of the confidence interval.

The prevalence of coronavirus-related PTSD, perceived stress anxiety, insufficient level of PA, depression, and suicidal/self-harm ideation during T1 and T2 are presented in Table 2 and Figure 2. H1 was fully confirmed. Pearson’s χ^2^ independence test was performed to compare the prevalence of coronavirus-related PTSD, perceived stress, anxiety, depression, suicidal ideation, and PA across the four countries. Each contingency table was examined separately for the particular mental health dimension for T1 and T2. Significant differences were found between countries in each mental health dimension during T1 and T2. However, the effect sizes were moderate for coronavirus-related PTSD during T2, PA during T1, and suicidal ideation during T1, whereas the effect sizes were small for the other dimensions. Young adults from Israel presented the lowest percent of anxiety in T1 and T2, coronavirus-related PTSD in T1 and T2, stress at T2, and an insufficient level of PA in T1 and T2, compared to the other countries. People from Germany showed the highest coronavirus-related PTSD during T1 and T2 and depression during T1 and T2, whereas individuals from Poland demonstrated the highest rate of perceived stress during T1 and T2 and risk of anxiety during T1 and T2. Insufficient levels of PA prevailed in Slovenia during T1 and T2 compared to the other countries. 

### 3.2. Change in Mental Health

**Hypothesis** **2** **(H2).** *There will be longitudinal changes in the mental health indicators among young adults across Germany, Israel, Poland, and Slovenia between T1 and T2.*

Longitudinal changes in mental health dimensions and PA between T1 and T2 of the COVID-19 pandemic were examined using McNemar’s χ^2^ (see Table 2 for details regarding the prevalence rate in the total sample). H2 was partially confirmed. A significant decrease of 5.17% (95% CI = 2.99%, 7.33%) in coronavirus-related PTSD risk was observed between T1 (65.47%) and T2 (60.30%) (McNemar’s χ^2^ = 21.47, *p* < 0.001). A significant improvement of approximately 4.88% (95% CI = 2.94%, 6.82%) in perceived stress was also found between T1 (83.28%) and T2 (78.41%) (McNemar’s χ^2^ = 24.16, *p* < 0.001). The results of McNemar’s χ^2^ test suggest that there is stability in anxiety scores between T1 (31.35%) and T2 (30.03%) (McNemar’s χ^2^ = 1.03, *p* = 0.31). The proportion of adults with an insufficient level of PA decreased by approximately 18.18% (95% CI = –0.63%, 35.96%), from 51.52% (T1) to 33.33% (T2), but these differences were insignificant (McNemar’s χ^2^ = 3.60, *p* = 0.06). Insignificant changes between T1 (34.76%) and T2 (34.36%) were also found in depression (McNemar’s χ^2^ = 0.13, *p* = 0.72) and suicidal ideation (McNemar’s χ^2^ = 0.13, *p* = 0.72): T1 = 36.33% and T2 = 36.74%. 

The GEE analysis was performed for coronavirus-related PTSD, perceived stress, anxiety, depression, and suicidal/self-harm ideation separately, including the other mental health indices as confounders (e.g., when a change in anxiety was examined, depression, PTSD, perceived stress, and PA were considered as covariates). The results of the GEE analysis are presented in Model 1 of Table 3. Changes in anxiety, depression, and suicidal ideation were not significant between T1 and T2. In contrast, a statistically significant decrease in the indices of the risk of coronavirus-related PTSD and perceived stress were observed between T1 and T2.

### 3.3. Sociodemographic Predictors of Change in Mental Health

**Hypothesis** **3** **(H3).** *Sociodemographic variables (country, gender, age, student status, employment status, relationship status, and having children) will be predictors of change in the mental health indicators among young adults during T1 and T2.*

The sociodemographic predictors of change in mental health are shown in Table 3 (Model 2). All sociodemographic variables were tested together in one model, separately for coronavirus-related PTSD, perceived stress, anxiety, depression, and suicidal ideation, including the other mental health indices as covariates.

Germany showed significantly higher coronavirus-related PTSD risk, whereas Israel presented a significantly lower rate than Poland and Slovenia. The other positive predictors of coronavirus-related PTSD were younger age (between 20 and 29 years old), being single, and having children. Significantly lower stress was predicted in Germany, Israel, and Slovenia than in Poland. Moreover, higher stress was predicted in women, single individuals, and those with children. When the country was considered a predictor of anxiety, depression, and suicidal ideation, significantly lower risks were expected in Israel and Slovenia compared to Poland. Furthermore, being single was found to be a predictor of higher anxiety, depression, and suicide risk. In addition, current student status was a predictor of a higher risk of depression and suicidal ideation.

## 4. Discussion 

In this study, we showed the prevalence of mental health indicators (coronavirus-related PTSD, perceived stress, anxiety, depression, and suicidal ideation) in each country and the changes in a total sample of 1723 young adults in a three-month period. We also revealed the risk factors among sociodemographic variables for mental health difficulties. 

The study was conducted with high methodological standards, including representative samples and longitudinal design in a cross-cultural context. Therefore, the results fulfill the criteria to be generalized to the young adult population.

### 4.1. Prevalence of Mental Health in the Four Countries

The results point to young adults in Israel as relatively low in mental health difficulties compared to the other countries. This provides a partial indication that Israeli youth demonstrate mental resilience. The population in Israel has been repeatedly exposed to stressors mainly related to political violence as part of the Israeli–Palestinian conflict. Therefore, many resilience centers offering mental health services and mental health intervention programs are available in the country; these may have promoted mental resilience on the individual and community levels [42,43]. Moreover, general health (including mental health) services are relatively accessible and affordable in Israel. In addition, the government in Israel offered long-term financial support to people who were laid off during the pandemic. This financial aid was given to young adults as well. In addition, Israel was among the first countries to make vaccinations available to all young adults. Furthermore, during the period of study, the most acute drop in a number of new cases of coronavirus per million between T1 and T2 was found in Israel compared to the other countries. Moreover, there was a drop in the severity of restrictions during T2. These can be factors that underline the relatively resilient response observed among the Israeli population. On the contrary, besides the relatively lower risk of coronavirus-related PTSD, anxiety, and depression, young adults in Israel reported the risk of high perceived stress, similar to other countries. 

Previous research among young adults with student status during the first wave of the pandemic has revealed the lowest prevalence of anxiety (5.20%) among German students compared to Poland, Israel, Slovenia, Czech Republic, Ukraine, Russia, Turkey, and Colombia [44]. In the present study, the anxiety prevalence among German young adults exceeded 30% in both measurements. However, these measurements were conducted between the second and third wave of the pandemic. In contrast, the prevalence of anxiety in Israeli students during the first wave was 38.70 [44], whereas in the present research, it did not exceed 20%. This shows a different pattern of mental health reaction to different waves of the pandemic in each country among similar groups of young adults.

It is noteworthy that the perceived stress levels are very high in each country (over 70%) in the present study. This means that, even though during T2 the pandemic restrictions were waived in most countries (except Germany) and the number of new COVID-19 cases dropped, the situation was still perceived as unstable and unpredictable. 

Each of the other countries was high on some of the mental health indicators compared to the other countries. In particular, Germany was relatively high in depression and coronavirus-related PTSD prevalence. Poland was high on anxiety and stress, whereas Slovenia was highest in terms of the prevalence of insufficient PA. The pandemic crisis manifested in a somewhat different pattern of mental health problems in each country. The culture, economy, political stability, and availability of health and support systems are factors that may explain the differences in the prevalence of mental health indicators between countries [45].

### 4.2. Mental Health Indicators over Time

The overall results demonstrate that several mental health indicators significantly improved over time during the unfolding of the pandemic, whereas other indicators remained stable. Specifically, coronavirus-related PTSD, stress, and insufficient PA decreased between T1 and T2, while depression, anxiety, and suicidal/self-harm ideation remained unchanged on average. 

This pattern of results is partly concurrent with recently published studies that followed changes in mental health indicators over time during the pandemic [15,16]. However, our research was conducted between the second and third waves of the COVID-19 pandemic, whereas previous studies have reported mental health outcomes from the first wave. Therefore, in this study, the risk of infection was lower and the pandemic restrictions were relatively low. If there is an interaction between mental health outcomes and the pandemic wave, the differences between previous studies and our studies are understandable.

Most previous studies reported a rise in mental health difficulties (anxiety, depression, and general mental health symptoms) during the first months of the pandemic; however, over several months, mental health indicators decreased or remained stable [15]. These trends also resemble changes in mental health symptoms following exposure to other natural disasters. For example, a study that examined mental health symptoms following Hurricane Sandy in 2012 in the U.S. reported significant decreases in anxiety scores and PTSD scores in a one-year follow-up after exposure, but no significant differences in depression were observed [46]. Thus, the rate of change of the metal health indicators may differ.

The findings provide evidence for mental adaptation and adjustment processes that take place. The pandemic brought about enormous changes in many aspects of people’s lives and it seems that over time, people have found ways to adjust to the new situation [1]. For example, at the beginning of the pandemic, the participants used to conduct their PA in gyms or other types of fitness centers, which, due to the risk of contamination, were closed for some time. In the following months, people found alternative ways to engage in PA. Such adaptations may promote an increase in PA across countries. In our results, the decrease in the prevalence of insufficient PA was close to significance (*p* = 0.06), but a clear trend was observed across all countries. Studies following PA activity during the pandemic thus far have mostly reported an overall decrease in PA [47,48]. The current findings show a trend of recovery in PA among young adults, which may contribute to mental health recovery.

Alongside the mental health indicators that showed improvement, anxiety, depression, and suicidal/self-harm ideation remained unchanged between T1 and T2. These specific mental health factors may take a more extended period of time for the adaptation processes to manifest. In a one-year follow-up following exposure to Hurricane Sandy (2012), PTSD and anxiety scores decreased, but depression scores did not [46]. Perhaps depression and suicidal/self-harm ideation are markers of more severe mental distress. Nonetheless, the current findings are a source of hope, showing mental health indicators to be stable rather than persistently rising. 

### 4.3. Sociodemographic Predictors of Mental Health

Examining sociodemographic variables as predictors of mental health yielded some interesting findings. Being single, having children, female gender, student status, and younger age (20–29 years) were found to be risk factors for some of the mental health indicators. On the contrary, male gender, being coupled, not being a student, and not having children were protective factors. 

These factors have been explored in past studies. A robust finding is that being single is associated with poorer well-being [49,50]. Recent research among Polish and American young adults also showed better mental health among partnered individuals [51]. However, that study revealed that satisfaction with one’s relationship status is a more powerful predictor than relationship status itself [51]. Additionally, it should be noted that there are stronger effects for relationships on mental health than vice versa [52]. However, in our longitudinal study of mental health among a representative population of young adults from the four countries, being single turned out to be the most common and strongest risk factor for all mental health outcomes. 

As for parenting, the burden of caring for children during a pandemic has been elevated, especially for those with young children. Studies have reported that parents’ mental health deteriorated during the pandemic, as schools were closed for periods of time and learning was conducted digitally from home for some time [53,54]. Women experienced more stress and distress during COVID-19 compared to men [55,56]. However, gender was not a significant predictor of coronavirus-related PTSD in our study, which is in congruence with previous research among young adults during the first wave of the pandemic [57,58]. In contrast, the recent research among university students from six countries during the second wave of the pandemic revealed gender to be a significant risk factor for coronavirus-related PTSD [59]. The differences in the role of gender can be explained by the group characteristics and the course of the pandemic.

Being a student is also a vulnerable state, as students have unique COVID-19-related concerns, such as rapid changes in academic studies, moving to online digital learning, working in unstable jobs, and losing a job during the pandemic. Previous research has shown that the biggest risk factor among concerns related to the pandemic for both anxiety and depression is the deterioration of students’ economic status. Additionally, in countries with stronger traditional family values (e.g., Poland and Russia), students’ concerns about the relationships with loved ones during the pandemic have also been shown to be risk factors for anxiety and depression [7]. The conditions of uncertainty brought about by the pandemic are critical for students in a developmental period that entails uncertainty and changes. Compared to adults aged 30–40 years, they often have less stability in their lives, thus making them more susceptible to uncertainty and crisis periods. In our study, being a student was a risk factor particularly for depression and suicidal/self-harm ideation. In the student population, the depression risk is higher than in the general population [44,45]. Students are also at a higher risk of depression than anxiety [44], in contrast to the general population [60].

These findings indicate that there are potentially vulnerable populations among young people that are more susceptible to the effects of the pandemic; hence, these populations need extended support and resources. These include young adults who are single, students, women, and parents. Policymakers will need to provide unique solutions for these groups with special consideration for their needs, including accessible mental health services.

### 4.4. Limitations

Despite the strengths of this study, which includes a longitudinal cross-national design, it has several limitations. Comparisons between countries should be examined with caution due to the differences in the COVID-19 situation in each country during the observed period, the extent of public health restrictions imposed by governments, and the related deaths in each of the examined countries. In addition, the study measures were exclusively based on self-reported measures. Thus, data can be subject to retrospective response bias. Previous research has shown discrepancies between self-reported depression symptoms and ratings by clinicians, especially among milder forms of depression [61]. Moreover, the sociodemographic variable of relationship status was highly simplistic and referred only to reporting being single versus being coupled. The single status itself can be described in at least 14 categories [62] and relationship status is a weaker predictor of mental health compared to satisfaction with relationship status [51].

It would be beneficial for future research to further follow mental health indicators over time and across countries, preferably using multisource measures, to examine whether adaptation and adjustment processes endure. 

## 5. Conclusions 

The importance of the current study is in examining the changes in mental health indicators among young adults over time during the pandemic across four countries. The results revealed that mental health remained stable and even improved for some indicators. These findings are encouraging and point to normative adaptation processes that young adults undergo when facing considerable strain. Some differences in the prevalence of mental health indicators were found between countries and these differences are likely due to health services, cultural, policy, and economic differences that may affect public health during the pandemic. Furthermore, vulnerability factors were revealed; these make some populations more susceptible to the effects of the pandemic on mental health, particularly single young adults. Future intervention programs for young adults should consider these factors in their prioritization, planning, and implementation.

## Figures and Tables

**Table 1:** Sociodemographic characteristics of the sample.

	Total (*n* = 1723)		Countries								Pearson’s	Cramer’s
			Germany (*n* = 418)		Israel (*n* = 428)		Poland (*n* = 446)		Slovenia (*n* = 431)			
Variable	*n*	%	*n*	%	*n*	%	*n*	%	*n*	%	χ^2^ (3)	V
**Gender**											5.85	0.06
Men	782	45.39	193	46.17	185	43.22	221	49.55	193	46.17		
Women	935	54.27	224	53.59	242	56.54	222	49.78	224	53.59		
**Age**											4.44	0.05
Adults	883	51.25	216	51.68	228	53.27	210	47.09	229	53.13		
Younger adults	840	48.75	202	48.33	200	46.73	236	52.92	202	46.87		
**Place of residence**											133.61 ***	0.28
Village	426	24.72	71	16.99	38	8.88	155	34.75	162	37.59		
Town or city	1297	75.28	347	83.01	390	91.12	291	65.25	269	62.41		
**Student status**											49.12 ***	0.17
No	1303	75.62	336	80.38	272	63.55	366	82.06	329	76.33		
Yes	420	24.38	82	19.62	156	36.45	80	17.94	102	23.67		
**Employment status**											9.46 *	0.07
Unemployed	399	23.16	91	21.77	92	21.50	93	20.85	123	28.54		
Employed	1324	76.84	327	78.23	336	78.51	353	79.15	308	71.46		
**Relationship status**											13.77 **	0.09
Coupled	1218	70.69	270	64.59	301	70.33	319	71.53	328	76.10		
Single	505	29.31	148	35.41	127	29.67	127	28.48	103	23.90		
**Having children**											23.47 ***	0.11
No	1001	58.10	273	65.31	232	54.21	228	51.12	268	62.18		
Yes	722	41.90	145	34.69	196	45.79	218	48.88	163	37.82		

Note. * *p* < 0.05, ** *p* < 0.01, and *** *p* < 0.001.

**Table 2:** Prevalence of mental health indices among young adults from Germany, Israel, Poland, and Slovenia in a three-month period.

	Total (*n* = 1723)		Countries								Pearson’s	Cramer’s
			Germany (*n* = 418)		Israel (*n* = 428)		Poland (*n* = 446)		Slovenia (*n* = 431)			
Variable	*n*	%	*n*	%	*n*	%	*n*	%	*n*	%	χ^2^ (3)	*V*
**COVID-19 PTSD T1**											64.86 ***	0.19
No risk	595	34.53	104	24.88	213	49.77	136	30.49	142	32.95		
Risk	1128	65.47	314	75.12	215	50.23	310	69.51	289	67.05		
**COVID-19 PTSD T2**											102.61 ***	0.24
No risk	684	39.70	117	27.99	255	59.58	149	33.41	163	37.82		
Risk	1039	60.30	301	72.01	173	40.42	297	66.59	268	62.18		
**Stress T1**											28.49 ***	0.13
Low	288	16.72	64	15.311	88	20.56	43	9.64	93	21.58		
High	1435	83.29	354	84.70	340	79.44	403	90.36	338	78.42		
**Stress T2**											28.26 ***	0.13
Low	372	21.59	88	21.053	116	27.10	60	13.45	108	25.06		
High	1351	78.41	330	78.95	312	72.890	386	86.55	323	74.94		
**Anxiety T1**											47.47 ***	0.17
No risk	887	51.48	263	62.92	345	80.61	279	62.56	347	80.51		
Risk	405	23.51	155	37.08	83	19.39	167	37.44	84	19.49		
**Anxiety T2**											47.47 ***	0.17
No risk	1242	72.08	286	68.42	342	79.91	276	61.88	338	78.42		
Risk	481	27.92	132	31.58	86	20.09	170	38.12	93	21.58		
**Physical activity T1**											76.72 ***	0.21
Sufficient	1003	58.21	248	59.33	297	69.39	280	62.78	178	41.230		
Insufficient	720	41.79	170	40.67	131	30.61	166	37.22	253	58.70		
**Physical activity T2**											33.44 ***	0.14
Sufficient	1175	68.20	299	71.53	314	73.36	316	70.85	246	57.08		
Insufficient	548	31.81	119	28.450	114	26.64	130	29.15	185	42.92		
**Depression T1**											55.60 ***	0.18
No risk	1124	65.24	235	56.22	315	73.60	255	57.18	319	74.01		
Risk	599	34.77	183	43.78	113	26.40	191	42.83	112	25.99		
**Depression T2**											54.67 ***	0.18
No risk	1131	65.64	239	57.18	302	70.56	258	57.85	332	77.03		
Risk	592	34.36	179	42.82	126	29.44	188	42.15	99	22.97		
**Suicidal Ideation T1**											81.16 ***	0.22
0 = No	1097	63.67	230	55.02	322	75.23	231	51.79	314	72.85		
1 = Yes	626	36.33	188	44.98	106	24.77	215	48.21	117	27.15		
**Suicidal Ideation T2**											64.37 ***	0.19
0 = No	1090	63.26	238	56.94	309	72.2	230	51.57	313	72.62		
1 = Yes	633	36.74	180	43.06	119	27.8	216	48.43	118	27.38		

Note. T1 = Time 1; T2 = Time 2; COVID-19 PTSD = coronavirus-related post-traumatic stress disorder; *** *p* < 0.001.

**Table 3:** Longitudinal predictors of coronavirus-related PTSD, perceived stress, anxiety, physical activity, depression, and suicidal ideation.

Variable	Coronavirus-Related PTSD			Stress			Anxiety			Depression			Suicidal/Self-Harm Ideation		
	B	OR	95% CI	B	OR	95% CI	B	OR	95% CI	B	OR	95% CI	B	OR	95% CI
Model 1: Change in mental health (vs. Time 1)															
Time 2	–0.22	0.80 ***	(0.73, 0.88)	–0.32	0.73 ***	(0.64, 0.83)	–0.02	0.98	(0.88, 1.09)	–0.02	0.98	(0.89, 1.08)	0.02	1.02	(0.92. 1.12)
**Model 2: Sociodemographic predictors of mental health change**															
Adjusted change (vs. Time 1)															
Time 2	–0.02	0.98	(0.67, 1.44)	–0.01	0.99	(0.58, 1.67)	0.03	1.03	(0.66, 1.59)	–0.06	0.94	(0.62, 1.43)	0.02	1.02	(0.92, 1.13)
Country (vs. Poland)															
Germany	–0.31	1.36 *	(1.00, 1.85)	–0.52	0.60 *	(0.39, 0.90)	–0.01	1.00	(0.75, 1.32)	0.02	1.02	(0.77, 1.35)	–0.20	0.82	(0.65, 1.04)
Israel	–0.83	0.44 ***	(0.33, 0.58)	–0.88	0.41 ***	(0.28, 0.62)	–0.95	0.39 ***	(0.28, 0.53)	–0.84	0.43 ***	(0.32, 0.58)	–1.03	0.36 ***	(0.28, 0.46)
Slovenia	–0.06	0.95	(0.71, 1.27)	–0.91	0.40 ***	(0.27, 0.60)	–0.89	0.41 ***	(0.30, 0.56)	–0.79	0.46 ***	(0.34, 0.61)	–0.90	0.41 ***	(0.32, 0.52)
Gender (vs. men)															
Women	0.08	1.08	(0.88, 1.33)	0.32	1.38 *	(1.06, 1.78)	0.15	1.16	(0.93, 1.45)	0.20	1.22	(0.99, 1.50)	–0.06	0.94	(0.79, 1.13)
Age (vs. adults)															
Younger Adults	0.28	1.33 *	(1.06, 1.67)	0.29	1.33	(0.99, 1.79)	0.14	1.15	(0.91, 1.47)	0.13	1.13	(0.90, 1.43)	0.11	1.12	(0.92, 1.35)
Student status (vs. No)															
Yes	0.14	1.15	(0.88, 1.49)	0.04	1.04	(0.74, 1.45)	0.25	1.23	(0.97, 1.71)	0.42	1.52 **	(1.17, 1.98)	0.32	1.37 ***	(1.10, 1.72)
Employment status (vs. unemployed)															
Employed	–0.12	0.88	(0.68, 1.15)	–0.10	0.91	(0.65, 1.27)	0.03	1.03	(0.78, 1.35)	–0.20	0.82	(0.63, 1.06)	0.16	1.17	(0.94, 1.47)
Relationship status (vs. coupled)															
Single	0.52	1.68 ***	(1.20, 2.19)	0.57	1.77 ***	(1.26, 2.48)	0.32	1.38 *	(1.05, 1.80)	0.55	1.73 ***	(1.34, 2.22)	0.44	1.55 ***	(1.24, 1.92)
Having children (vs. No)															
Yes	0.30	1.35 *	(1.06, 1.72)	0.32	1.37 *	(1.01, 1.86)	0.24	1.28	(0.99, 1.65)	0.21	1.23	(0.96, 1.58)	0.03	1.03	(0.84, 1.27)

Note. PTSD = post-traumatic stress disorder; AOR = adjusted odds ratio; CI—confidence interval. * *p* < 0.05, ** *p* < 0.01, and *** *p* < 0.001.

## Data Availability

This study is a part of an international research project “Mental health of young adults during the COVID-19 pandemic in Poland, Germany, Slovenia and Israel: a longitudinal study” [63], registered at the Center for Open Science (OSF). The datasets used and analyzed during the current study are available from the corresponding author upon reasonable request.

## Supplementary Materials

The following supporting information can be downloaded at: https://www.mdpi.com/article/10.3390/ijerph19031334/s1, Table S1: The number of participants regarding the place of residence in Germany, Israel, Slovenia, and Poland.
