# Supplementary material for: Prevalence and Sociodemographic Predictors of Mental Health in a Representative Sample of Young Adults from Germany, Israel, Poland, and Slovenia: A Longitudinal Study during the COVID-19 Pandemic
Source: Int J Environ Res Public Health. 2022 Jan 25;19(3):1334. doi: 10.3390/ijerph19031334 (PMC8835083; doi:10.3390/ijerph19031334)
Supplement: Supplementary file 1 [file ijerph-19-01334-s001.zip › ijerph-1552771-supplementary.pdf]

Supplementary Table S1

The number of participants regarding the place of residence in Germany, Israel, Slovenia, and Poland.

Country	District / Voivodship	*N*	%
Germany	Baden-Württemberg	50	12.0
	Bayern	62	14.8
	Berlin	17	4.1
	Brandenburg	11	2.6
	Hessen-Thüringen	45	10.8
	Mecklenburg-Vorpommern	8	1.9
	Niedersachsen-Bremen	46	11.0
	Nordrhein-Westfalen	92	22.0
	Rheinland-Pfalz-Saarland	22	5.3
	Sachsen- Sachsen-Anhalt	39	9.3
	Schleswig-Holstein-Hamburg	26	6.2
	Total	418	100
Israel	Northern District	50	11.7
	Haifa District	51	11.9
	Central District	132	30.8
	Tel Aviv District	87	20.3
	Jerusalem District	44	10.3
	Southern District	54	12.6
	From the Judea and Samaria District	10	2.3
	Total	428	100.0
Slovenia	East Slovenia	242	56.10
	West Slovenia	189	43.90
	Total	431	100
Poland	Dolnośląskie (Lower Silesia)	28	6.3
	Kujawsko-Pomorskie (Kuyavia-Pomerania)	17	3.8
	Lubelskie (Lublin)	40	9.0
	Lubuskie	12	2.7
	Łódzkie (Lodzkie)	25	5.6
	Małopolskie (Lesser Poland)	25	5.6
	Mazowieckie (Masovia)	72	16.1
	Opolskie (Opole)	19	4.3
	Podkarpackie (Subcarphatia)	37	8.3
	Podlaskie	12	2.7
	Pomorskie (Pomerania)	19	4.3
	Śląskie (Silesia)	49	11.0
	Świętokrzyskie	24	5.4
	Warmińsko-Mazurskie (Warmia-Masuria)	12	2.7
	Wielkopolska (Greater Poland)	41	9.2
	Zachodniopomorskie (West Pomerania)	14	3.1
	Total	446	100
TOTAL		1,723	100
